# Diagnostic testing in the genetically complex age-related macular degeneration

**DOI:** 10.1515/medgen-2024-2064

**Published:** 2025-02-12

**Authors:** Christina Kiel, Bernhard H. F. Weber

**Affiliations:** University of Regensburg Institute of Human Genetics Franz-Josef-Strauss-Allee 11 93053 Regensburg Germany; University of Regensburg Institute of Human Genetics Franz-Josef-Strauß-Allee 11 93053 Regensburg Germany

**Keywords:** Age-related macular degeneration, AMD, DNA diagnostics, risk prediction, polygenic risk score

## Abstract

Age-related macular degeneration (AMD) is a leading cause of visual impairment with the risk of developing the disease influenced by a combination of genetic and environmental factors. With the recent expansion of treatment options, enhancing diagnostic accuracy and improving access to treatment are increasingly becoming the focus of interest. By using data from genome-wide association studies (GWAS) to generate polygenic risk scores (PRS), an assessment of an individual’s genetic risk for AMD is feasible. While the predictive accuracy of the AMD-PRS is most robust for individuals at very high genetic risk, genetic diagnostic testing is warranted due to the large number of affected individuals resulting from the high prevalence of AMD. Early genetic confirmation of AMD-related pathology can facilitate timely treatment initiation, potentially improving patient outcomes.

## Introduction

Blindness and moderate to severe vision impairment (MSVI) constitute a significant burden to patients, the worldwide health care systems, and the economy. A recent review, based on data from 2020, estimated the global annual productivity loss due to blindness and MSVI at around $410.7 billion [1]. In Europe, 2.6 million people are predicted to be affected by blindness, and an additional 30.5 million by MSVI [2]. In the population aged 50 years and older, age-related macular degeneration (AMD) is one of the main causes of blindness and MSVI, along with cataract, insufficiently corrected refractive error, and glaucoma [3]. In comparison to other major contributors to blindness and MSVI, treating and controlling the progression of AMD is challenging. Despite the complexities, new therapeutic options for AMD have recently become available but therapy is mainly considered effective if initiated early in the disease process. This article explores what information a genetic test for AMD can provide and examines the scenarios in which such testing might be beneficial.

## Therapeutic options for AMD

As AMD is a leading cause of blindness and MSVI among the elderly, possible treatment options are of major importance for affected individuals. The late stages of AMD can manifest in two forms, the more frequent geographic atrophy and the neovascular complication. These two forms can develop separately or concurrently in the same eye.

Geographic atrophy is characterized by distinct areas of depigmentation, with important hallmarks being a functional loss of the retinal pigment epithelium and a causative involvement of the innate immune system, specifically the complement system. This form of AMD generally progresses slowly over a few years [4,5].

Neovascular AMD involves the disruption of Bruch’s membrane, retinal pigment epithelium, and photoreceptors by newly formed choroidal vessels. Such vessels are fragile and leak blood and fluid, leading to subretinal hemorrhage, fibrous scarring, and retinal detachment. This form of AMD progresses more rapidly and can cause significant vision loss in days and weeks rather than years [4,5].

For neovascular AMD, the first anti-vascular endothelial growth factor (VEGF) therapy ranibizumab (Lucentis) was approved by the FDA in 2006 [6], and by the EMA in 2007 [7]. VEGF plays an important role in angiogenesis and favors the formation of new blood vessels. Anti-VEGF drugs inhibit VEGF, thereby reducing the growth and leakage of abnormal blood vessels in neovascular AMD. Since 2006, several anti-VEGF therapies have been introduced to the market [8].

In February 2023, the FDA approved pegcetacoplan (SYFOVRE™) as the first therapy for geographic atrophy due to AMD [9]. Pegcetacoplan targets the C3 complement protein, thus inhibiting the downstream effects of an overactivated complement system [10]. Surprisingly, European approval for pegcetacoplan was denied by the EMA. While it was accepted that SYFOVRE™ treatment reduces lesion size in patients with geographic atrophy, it was criticized that treatment fails to improve visual function after 24 months of therapy [10,11]. Consequently, the EMA concluded that the treatment offers no significant clinical benefits to patients [11].

Another treatment for geographic atrophy, known as avacincaptad pegol (IZERVAY™), was approved by the FDA in August 2023 [12]. This therapeutic approach also targets the complement system, specifically acting as an inhibitor of complement C5 proteins [13]. A decision by the EMA regarding IZERVAY™ treatment for the European market is still pending.

## Addressing complex diseases like AMD by genetic testing

AMD is classified as a complex disease. While for many decades, clinical genetic testing is routinely applied for monogenic diseases using DNA sequencing technologies such as Sanger sequencing and next-generation sequencing, complex diseases result from the interplay of multiple genetic and environmental factors, making the diagnostic process more demanding. In AMD, age and genetic predisposition are well established as major contributors to disease [3,14]. Additionally, other factors, such as gender, smoking, ethnicity, and diet, are still in discussion [3,14–17].

The genetic components of complex diseases generally involve numerous genomic regions, which often cannot be assigned to a particular gene, while each factor usually contributes only a small effect. Due to the interplay of multiple genetic factors, environmental influences, and the typically late onset of complex diseases, prognostic testing based purely on genetic factors has only limited utility. Genetic DNA testing using methods such as gene panel or whole exome/whole genome sequencing can be applied to exclude the presence of known monogenic disorders phenotypically mimicking AMD. Once a monogenetic condition is ruled out, further testing can be conducted to explore the contribution of genetic markers associated with the complex disease of interest and to define an individual genetic risk profile of a patient. A genetic risk profile is often quantified as a polygenic risk score (PRS), which can be used for diagnostic purposes. The diagnostic performance of a PRS – defined as its ability to determine whether a person is affected by the disease in question – varies significantly depending on the disease. In general, PRS tend to perform better in complex diseases with higher heritability [18]. AMD has a heritability estimate of 46.7 % [19], with some estimates reaching up to 71 % [20], which is in the medium to upper range for the heritability estimates of complex diseases [18]. It is important to note that the use of PRS for diagnostic purposes in Germany is currently limited to research settings and is not part of accredited diagnostic procedures. PRS may also have prognostic applications when combined with clinical scores, which have shown prognostic value in conditions such as breast cancer and cardiovascular disease [21,22]. However, in the case of AMD, such a combination is not currently feasible, as there are no established clinical measures that can be integrated into this framework.

## Risk assessment – the role of GWAS, PRS and machine learning in AMD

Genome-wide association studies (GWAS) are a powerful tool for investigating the genetic contributions to complex diseases, crucial for enabling risk assessment in conditions such as AMD. GWAS are designed to identify associations between genetic variants and disease traits by comparing allele frequencies between patients and healthy controls. The results from GWAS can be utilized to determine an individual’s aggregate genetic risk through the calculation of PRS.

A straightforward and common method to assess the PRS involves first determining the patient’s genotype for genetic variants identified by GWAS. The variants present in the patient are then weighted by their respective effect sizes and summed up. By comparing the resulting score with scores obtained from a reference population, an estimate of the individual’s genetic risk can be made. This approach allows PRS to provide a cumulative risk assessment based on the genetic profile of the individual [23].

Among the numerous genetic variants identified to be associated with a complex disease, a few exert strong effect sizes that significantly drive the disease risk. For example, AMD-associated variants in the *CFH* gene locus and the *ARMS2*/*HTRA1* locus are known to have substantial impact [19]. Consequently, while weaker effect variants contribute to the overall PRS, their individual exclusion is not critical as their collective influence is relatively minor [23]. However, it is crucial to ensure that variants with strong effect sizes are accurately included in the PRS calculations. If such a variant fails quality control measures and is omitted, the resulting risk score may lose its predictive power and reliability.

Over time, more statistically sophisticated and complex methods have been developed for calculating a PRS. These methods include considerations of linkage disequilibrium (LD), which assesses whether two genetic variants co-occur more or less frequently than expected. One such method is LD clumping and thresholding, which considers the LD of variants that pass a specific p-value threshold, effectively filtering out redundant signals [24]. However, the identification of independent signals and the consideration of LD structures are typically already addressed in most GWAS, including the AMD GWAS conducted by Fritsche and colleagues in 2016 [19]. This approach, for instance, allowed the resolution of the signal at the *CFH* locus, which is strongly associated with AMD, into eight independent signals.

With the increasing amount of GWAS data and the corresponding rise in identified genetic associations – often with small to very small effect sizes – alternative approaches to traditional GWAS-based PRS are gaining popularity. One such approach involves Bayesian selection methods, such as LDpred or PRS-CS (continuous shrinkage). These methods incorporate LD information from a reference panel and GWAS summary statistics to estimate posterior mean causal effect sizes [25,26]. Another innovative approach is the use of machine learning-optimized PRS, which selects genetic variants from a training dataset rather than from GWAS results, aiming to maximize predictive power [27]. For example, in a recent multi-ancestry GWAS for rheumatoid arthritis, 124 loci associated with the disease were identified [28]. Traditional GWAS-based PRS, would typically use all 124 loci [28]. However, a machine learning-optimized PRS demonstrated that strong predictive power could be achieved using only nine genetic variants for rheumatoid arthritis [27]. This highlights how advanced PRS methods, such as those optimized through machine learning, offer a promising new alternative for genetic diagnostics. By effectively reducing the number of genetic variants needed for accurate prediction, these methods enhance the feasibility and applicability of genetic testing for complex diseases in clinical settings.

Additionally, machine learning can significantly enhance the interpretability and utility of PRS. Contemporary methods combine PRS with additional factors such as patient age and gender, to predict disease risk more accurately [29]. This approach not only improves the predictive power of PRS but also provides a more comprehensive and personalized risk assessment.

## Applicability and interpretability of genetic diagnostics for AMD

Following, the applicability and interpretability of genetic diagnostic testing specifically for AMD is discussed. For establishment and validation data from the International AMD Genomics Consortium (IAMDGC) were used [19]. A PRS was calculated on the basis of 47 out of the 52 AMD-associated independent variants described by Fritsche and colleagues in 2016 [19]. Due to low coverage in the panel used for diagnostic testing the following variants were excluded: rs187328863 (one out of eight independent signals in the *CFH* locus, which does not correspond to the main signal with the lead variant rs10922109 of the *CFH* locus, odds ratio (OR) = 1.47 as described in [19]), rs114092250 (*PRLR*/*SPEF2* locus, OR = 0.71), rs10781182 (*MIR6130*/*RORB* locus, OR = 1.12), rs67538026 (*CNN2* locus, OR = 0.9) and rs201459901 (*C20orf85* locus, OR = 0.76). In fact, not all lead variants can be easily genotyped in a sufficient quality. This was noted earlier by the EYE-RISK Consortium while developing a genotype assay for AMD in 2021 [30].

A weighted GWAS-based model was used to determine the PRS [23]. The risk alleles of the respective genetic variants were counted and multiplied by their effect size. The weighted risk alleles were summed and divided by the mean effect size of all AMD risk variants. This way a PRS is obtained for each person, which reflects the number of risk alleles with an average effect size for the person. In addition, a machine learning based algorithm, namely Mondrian Cross-Conformal Prediction, was applied to determine the AMD disease status based on the PRS, age and gender of the patient [29]. Individuals from European ancestry in the Regensburg cohort of the IAMDGC dataset were used in the training dataset, including 1,667 patients with a late-stage form of AMD and 1,148 controls. The remaining European individuals from the IAMDGC dataset, including 14,209 late-stage AMD patients and 16,566 controls, were included for validation [19].

**Figure 1:**
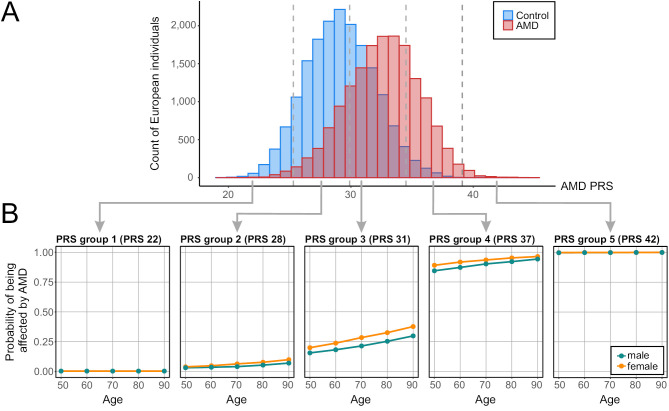
**Polygenic risk score (PRS) distribution and predictive expressiveness. (A)** PRS distribution of patients with age-related macular degeneration (AMD) and healthy controls of European descent from the International AMD Genomics Consortium (IAMDGC) [19]. The PRS was calculated using 47 out of 52 independent genome-wide association study (GWAS) lead variants [19], representing the cumulative genetic risk of each individual. Risk quintiles are indicated by gray dashed lines and are defined as follows: group 1 PRS ≤ 25.3, group 2 25.31 – 29.9, group 3 29.91 – 34.5, group 4 34.51 – 39.1, and group 5 > 39.1. Overall, AMD patients exhibit a genetic risk profile shifted towards a higher genetic risk compared to healthy controls. **(B)** A machine learning approach was used for a disease status prediction based on PRS, age and gender [29]. Individuals from the IAMDGC dataset recruited in Regensburg were used as a training dataset. A valid disease prediction is feasible for individuals with either a very high (group 5) or very low (group 1) PRS.

When constructing the PRS for a cohort, the scores typically follow a Gaussian distribution. Generally, AMD patients exhibit a slightly higher average risk compared to unaffected individuals (Figure 1A), although there is substantial overlap between the two distributions. To enhance interpretability, one effective strategy is to classify individuals into risk groups, such as quintiles, based on their PRS [23]. This classification assigns people to specific risk categories, making it possible to interpret disease risk more precisely. Predictive statements about disease status are most meaningful for individuals in the very low or very high-risk groups (Figure 1B), although additional factors like gender and age are considered. For those in the medium-risk categories, no sufficient prediction of the disease status is possible.

**Figure 2:**
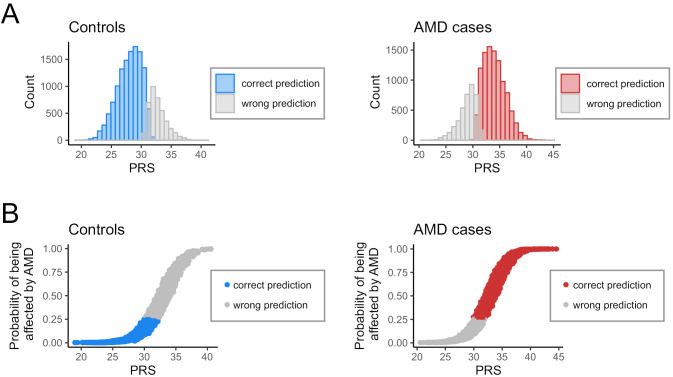
**Validation of the disease prediction model for AMD. (A)** PRS distribution of correctly and incorrectly predicted AMD cases and controls using the machine learning approach. **(B)** Probability of being affected by AMD for correctly and incorrectly predicted AMD cases and controls, displayed according to PRS.

This pattern is also evident in the validation of the prediction model. The overall sensitivity of the prediction model was 72.1 % and the overall specificity was 75.7 %. There is a reliable prediction range for both AMD cases and controls at the extremes (Figure 2A), but accuracy diminishes considerably in the overlapping region. This is also reflected in the predicted probability of being affected, which is made possible by the application of the machine learning approach (Figure 2B). While individuals with a very low or very high genetic risk are reliably predicted as being unaffected or affected, respectively, the probability for the middle risk groups lies in a range between around 20 and 80 % and therefore allows only limited conclusions.

**Figure 3:**
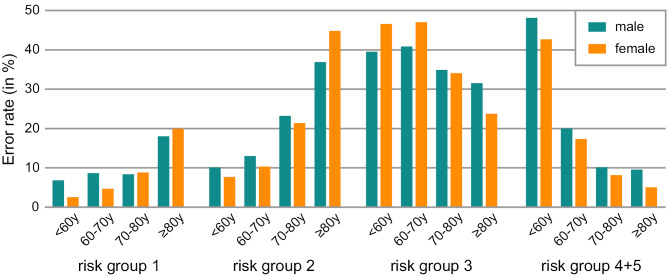
**Error rates of disease status prediction by risk group.** The percentages of incorrectly predicted individuals are shown per risk group, age and gender.

This relationship between genetic risk and conclusive prediction is additionally reflected in the error rates (Figure 3), which vary substantially between the different risk groups. For individuals in the middle risk groups (2 and 3), the average error across both genders and all age groups is around 29 %, indicating less reliable predictions for these groups. In the low-risk group (group 1), predictions are reasonably solid, particularly for individuals up to the age of 80, with an error rate below 10 %. However, for those older than 80, non-genetic factors including age seem to have a stronger influence, increasing the error rate to approximately 20 %. In the highest risk groups (4 and 5), predictions for people under 60 years of age are rather erroneous, with an error rate of approximately 45 %. However, as age increases, the reliability of predictions improves significantly, with the error rate dropping to 10 % and below for individuals over the age of 70 years. It should be noted that the dataset was divided into several smaller subsets to determine the error rates, resulting in sample sizes within separate groups ranging from 73 to 2,862 individuals.

There is no noticeable difference in the error rate between the two genders for the individual age and risk groups. It should be noted that this method is designed to assess whether a person is currently affected by AMD, rather than to predict future disease onset, though such projections can be inferred.

In considering the application of genetic diagnostics for AMD, it is important to determine the percentage of the population for which a valid risk assessment can be made based on genetic profiles. In 2012, Grassmann and colleagues demonstrated that approximately 0.5 % of the European population falls into the highest risk quintile for AMD, with an additional 9 % in the second highest risk quintile, which already provides relatively good predictive values [23]. However, genetic diagnostics would primarily be used for individuals who already exhibit symptoms of the disease. Analysis of the PRS distribution in the IAMDGC dataset shows that 27 % of late-stage AMD patients are represented in the two highest quintiles. Given that AMD is a frequent complex disease, it is estimated that around 300,000 people in Central and Eastern Europe and Central Asia were either blind or had MSVI due to AMD in 2020 [3]. Based on these numbers, approximately 81,000 individuals fall into the highest two risk quintiles. This expressive number underscores the potential impact of applying genetic diagnostics to identify high-risk individuals and facilitate early intervention and management.

## Limitations

Ethnicity is a critical factor in the applicability of PRS derived from GWAS or even for machine learning optimized PRS approaches. For instance, Fritsche and colleagues predominantly included participants of European descent in the AMD GWAS conducted in 2016 [19]. Since common genetic variants often exhibit significant variability between populations, the PRS derived from this GWAS can only provide accurate risk assessments for individuals with a European background. In case of machine learning optimized PRS approaches, the validity heavily depends on the ethnic composition of the training dataset. To address this limitation, one potential approach can be to preprocess GWAS summary statistics from multiple ethnicities using a weighted meta-analysis before forming a PRS [31].

Additionally, the smoking status of individuals was not included in the approach described here. Although smoking is a well-known risk factor for AMD [15], the self-reported smoking data from study participants and patients is often biased [32]. Including smoking status could enhance the accuracy of risk and disease status assessments.

A significant limitation of the PRS results presented here for AMD is that the same dataset was used for both the machine learning performance estimation and the original GWAS. This lack of independence between datasets can lead to an overestimation of predictive performance [33,34]. Koch and colleagues highlighted in a recent review on the clinical applicability of PRS [18] that few PRS have been validated using independent datasets and that the observed predictive power is typically lower in external validation studies than in the initial findings. Consequently, the predictive power of the PRS for AMD, as evaluated in the validation steps conducted in this study, is likely overestimated. To gain a more accurate assessment of the diagnostic, and potentially even prognostic, utility of a PRS for AMD, independent long-term follow-up studies are needed.

Finally, a significant challenge for the implementation of genetic tests is patient counseling. It is crucial that patients are thoroughly informed about the meaningfulness of diagnostic tests and the potential benefits they offer. It is crucial to distinguish clearly between diagnostic and prognostic tests for patients. Preventive treatment with AMD medications, particularly intravitreal injections, should be approached with caution due to the associated discomfort and side effects. The adverse effects of intravitreal injections, which do not provide significant clinical benefits, were a major reason for the EMA’s decision not to approve SYFOVRE™ for the European market [11]. Understanding the potential benefits and risks is essential for the effective application of genetic diagnostic testing for complex diseases in clinical practice. One way to enhance collaboration between human geneticists and clinical ophthalmologists, and to optimize patient care, is through the establishment of genetic diagnostic boards. These boards can discuss and evaluate the individual value of genetic testing for a patient, especially in cases of uncertainty, integrating it with clinical diagnoses to ensure a comprehensive approach to patient management.

## Conclusion/Summary

Genetic diagnostic testing for complex diseases such as AMD is not recommended as a standalone prognostic tool due to the intricate interplay between genetic and environmental factors. Notably, current genetic tests for AMD do not adequately account for environmental risk factors, which significantly limits their prognostic utility. However, once clinical symptoms of AMD manifest, genetic testing may provide additional value for an estimated 9.5 % to 27 % of patients. These figures are projections, and the actual values likely fall somewhere in between. Validation through independent long-term studies is essential to confirm these estimates.

While there is no cure for AMD, early treatment initiation can slow disease progression, which is crucial given the substantial visual impairment and associated comorbidities such as depression experienced by AMD patients [35,36].

Despite these benefits, there are technical limitations to consider. Most genetic studies in the past mainly involved European individuals, making it challenging to generalize findings across ethnic populations. Nonetheless, the application of advanced methods, such as machine learning, is expected to enhance the applicability and accuracy of genetic tests for complex diseases in the future, potentially overcoming current limitations and improving patient outcomes.

## References

[1] Burton MJ, Ramke J, Marques AP, Bourne RR, Congdon N, Jones I, Ah Tong BA, Arunga S, Bachani D, Bascaran C, Bastawrous A, Blanchet K, Braithwaite T, Buchan JC, Cairns J, Cama A, Chagunda M, Chuluunkhuu C, Cooper A, Crofts-Lawrence J, Dean WH, Denniston AK, Ehrlich JR, Emerson PM, Evans JR, Frick KD, Friedman DS, Furtado JM, Gichangi MM, Gichuhi S, Gilbert SS, Gurung R, Habtamu E, Holland P, Jonas JB, Keane PA, Keay L, Khanna RC, Khaw PT, Kuper H, Kyari F, van Lansingh C, Mactaggart I, Mafwiri MM, Mathenge W, McCormick I, Morjaria P, Mowatt L, Muirhead D, Murthy GV, Mwangi N, Patel DB, Peto T, Qureshi BM, Salomão SR, Sarah V, Shilio BR, Solomon AW, Swenor BK, Taylor HR, Wang N, Webson A, West SK, Wong TY, Wormald R, Yasmin S, Yusufu M, Silva JC, Resnikoff S, Ravilla T, Gilbert CE, Foster A, Faal HB (2021). The Lancet Global Health Commission on Global Eye Health: vision beyond 2020. The Lancet. Global health 9.

[2] (2021). Trends in prevalence of blindness and distance and near vision impairment over 30 years: an analysis for the Global Burden of Disease Study. The Lancet. Global health 9.

[3] (2021). Causes of blindness and vision impairment in 2020 and trends over 30 years, and prevalence of avoidable blindness in relation to VISION 2020: the Right to Sight: an analysis for the Global Burden of Disease Study. The Lancet. Global health 9.

[4] Swaroop A, Chew EY, Rickman CB, Abecasis GR (2009). Unraveling a multifactorial late-onset disease: from genetic susceptibility to disease mechanisms for age-related macular degeneration. Annual review of genomics and human genetics 10.

[5] van Lookeren Campagne M, LeCouter J, Yaspan BL, Ye W (2014). Mechanisms of age-related macular degeneration and therapeutic opportunities. The Journal of pathology 232.

[6] (2024). Drug Approval Package: Lucentis (Ranibizumab) NDA #125156. Drug Approval Package: Lucentis (Ranibizumab) NDA #125156.

[7] (2024). Lucentis. Lucentis.

[8] Song D, Liu P, Shang K, Ma Y (2022). Application and mechanism of anti-VEGF drugs in age-related macular degeneration. Frontiers in bioengineering and biotechnology 10.

[9] (2024). FDA Approves SYFOVRE™ (pegcetacoplan injection) as the first and only treatment for geographic atrophy (GA), a leading cause of blindness. FDA Approves SYFOVRE™ (pegcetacoplan injection) as the first and only treatment for geographic atrophy (GA), a leading cause of blindness.

[10] Heier JS, Lad EM, Holz FG, Rosenfeld PJ, Guymer RH, Boyer D, Grossi F, Baumal CR, Korobelnik J-F, Slakter JS, Waheed NK, Metlapally R, Pearce I, Steinle N, Francone AA, Hu A, Lally DR, Deschatelets P, Francois C, Bliss C, Staurenghi G, Monés J, Singh RP, Ribeiro R, Wykoff CC (2023). Pegcetacoplan for the treatment of geographic atrophy secondary to age-related macular degeneration (OAKS and DERBY): two multicentre, randomised, double-masked, sham-controlled, phase 3 trials. The Lancet 402.

[11] (2024). Syfovre. Syfovre.

[12] (2024). Iveric Bio Receives U. S. FDA Approval for IZERVAY™ (avacincaptad pegol intravitreal solution), a New Treatment for Geographic Atrophy. Iveric Bio Receives U. S. FDA Approval for IZERVAY™ (avacincaptad pegol intravitreal solution), a New Treatment for Geographic Atrophy.

[13] Khanani AM, Patel SS, Staurenghi G, Tadayoni R, Danzig CJ, Eichenbaum DA, Hsu J, Wykoff CC, Heier JS, Lally DR, Monés J, Nielsen JS, Sheth VS, Kaiser PK, Clark J, Zhu L, Patel H, Tang J, Desai D, Jaffe GJ (2023). Efficacy and safety of avacincaptad pegol in patients with geographic atrophy (GATHER2): 12-month results from a randomised, double-masked, phase 3 trial. Lancet.

[14] (2024). Global estimates on the number of people blind or visually impaired by age-related macular degeneration: a meta-analysis from 2000 to 2020. Eye 38.

[15] Delcourt C, Diaz JL, Ponton-Sanchez A, Papoz L (1998). Smoking and age-related macular degeneration. The POLA Study. Pathologies Oculaires Liées à l’Age. Archives of ophthalmology 116.

[16] Colijn JM, Meester-Smoor M, Verzijden T, Breuk A de, Silva R, Merle BM, Cougnard-Grégoire A, Hoyng CB, Fauser S, Coolen A, Creuzot-Garcher C, Hense H-W, Ueffing M, Delcourt C, Hollander AI den, Klaver CC (2021). Genetic risk, lifestyle, and age-related macular degeneration in Europe: The EYE-RISK Consortium. Ophthalmology 128.

[17] Angelia M, Amelia YS, Pratama KG (2024). Mediterranean diet as a modifiable risk factor for age-related macular degeneration: A systematic review and meta-analysis. Tzu chi medical journal 36.

[18] Koch S, Schmidtke J, Krawczak M, Caliebe A (2023). Clinical utility of polygenic risk scores: a critical 2023 appraisal. Journal of community genetics 14.

[19] Fritsche LG, Igl W, Bailey JN, Grassmann F, Sengupta S, Bragg-Gresham JL, Burdon KP, Hebbring SJ, Wen C, Gorski M, Kim IK, Cho D, Zack D, Souied E, Scholl HP, Bala E, Lee KE, Hunter DJ, Sardell RJ, Mitchell P, Merriam JE, Cipriani V, Hoffman JD, Schick T, Lechanteur YT, Guymer RH, Johnson MP, Jiang Y, Stanton CM, Buitendijk GH, Zhan X, Kwong AM, Boleda A, Brooks M, Gieser L, Ratnapriya R, Branham KE, Foerster JR, Heckenlively JR, Othman MI, Vote BJ, Liang HH, Souzeau E, McAllister IL, Isaacs T, Hall J, Lake S, Mackey DA, Constable IJ, Craig JE, Kitchner TE, Yang Z, Su Z, Luo H, Chen D, Ouyang H, Flagg K, Lin D, Mao G, Ferreyra H, Stark K, Strachwitz CN von, Wolf A, Brandl C, Rudolph G, Olden M, Morrison MA, Morgan DJ, Schu M, Ahn J, Silvestri G, Tsironi EE, Park KH, Farrer LA, Orlin A, Brucker A, Li M, Curcio CA, Mohand-Saïd S, Sahel J-A, Audo I, Benchaboune M, Cree AJ, Rennie CA, Goverdhan SV, Grunin M, Hagbi-Levi S, Campochiaro P, Katsanis N, Holz FG, Blond F, Blanché H, Deleuze J-F, Igo RP, Truitt B, Peachey NS, Meuer SM, Myers CE, Moore EL, Klein R, Hauser MA, Postel EA, Courtenay MD, Schwartz SG, Kovach JL, Scott WK, Liew G, Tan AG, Gopinath B, Merriam JC, Smith RT, Khan JC, Shahid H, Moore AT, McGrath JA, Laux R, Brantley MA, Agarwal A, Ersoy L, Caramoy A, Langmann T, Saksens NT, Jong EK de, Hoyng CB, Cain MS, Richardson AJ, Martin TM, Blangero J, Weeks DE, Dhillon B, van Duijn CM, Doheny KF, Romm J, Klaver CC, Hayward C, Gorin MB, Klein ML, Baird PN, Hollander AI den, Fauser S, Yates JR, Allikmets R, Wang JJ, Schaumberg DA, Klein BE, Hagstrom SA, Chowers I, Lotery AJ, Léveillard T, Zhang K, Brilliant MH, Hewitt AW, Swaroop A, Chew EY, Pericak-Vance MA, DeAngelis M, Stambolian D, Haines JL, Iyengar SK, Weber BH, Abecasis GR, Heid IM (2016). A large genome-wide association study of age-related macular degeneration highlights contributions of rare and common variants. Nature genetics 48.

[20] Seddon JM, Cote J, Page WF, Aggen SH, Neale MC (2005). The US twin study of age-related macular degeneration: relative roles of genetic and environmental influences. Archives of ophthalmology (Chicago, Ill.: 1960) 123.

[21] Lee A, Mavaddat N, Wilcox AN, Cunningham AP, Carver T, Hartley S, Babb de Villiers C, Izquierdo A, Simard J, Schmidt MK, Walter FM, Chatterjee N, Garcia-Closas M, Tischkowitz M, Pharoah P, Easton DF, Antoniou AC (2019). BOADICEA: a comprehensive breast cancer risk prediction model incorporating genetic and nongenetic risk factors. Genetics in medicine: official journal of the American College of Medical Genetics 21.

[22] O’Sullivan JW, Raghavan S, Marquez-Luna C, Luzum JA, Damrauer SM, Ashley EA, O’Donnell CJ, Willer CJ, Natarajan P (2022). Polygenic Risk Scores for Cardiovascular Disease: A Scientific Statement From the American Heart Association. Circulation 146.

[23] Grassmann F, Fritsche LG, Keilhauer CN, Heid IM, Weber BH (2012). Modelling the genetic risk in age-related macular degeneration. PloS one 7.

[24] Privé F, Vilhjálmsson BJ, Aschard H, Blum MG (2019). Making the Most of Clumping and Thresholding for Polygenic Scores. American journal of human genetics 105.

[25] Vilhjálmsson BJ, Yang J, Finucane HK, Gusev A, Lindström S, Ripke S, Genovese G, Loh P-R, Bhatia G, Do R, Hayeck T, Won H-H, Kathiresan S, Pato M, Pato C, Tamimi R, Stahl E, Zaitlen N, Pasaniuc B, Belbin G, Kenny EE, Schierup MH, Jager P de, Patsopoulos NA, McCarroll S, Daly M, Purcell S, Chasman D, Neale B, Goddard M, Visscher PM, Kraft P, Patterson N, Price AL (2015). Modeling Linkage Disequilibrium Increases Accuracy of Polygenic Risk Scores. American journal of human genetics 97.

[26] Ge T, Chen C-Y, Ni Y, Feng Y-CA, Smoller JW (2019). Polygenic prediction via Bayesian regression and continuous shrinkage priors. Nature communications 10.

[27] Lim AJ, Tyniana CT, Lim LJ, Tan JW, Koh ET, Chong SS, Khor CC, Leong KP, Lee CG (2023). Robust SNP-based prediction of rheumatoid arthritis through machine-learning-optimized polygenic risk score. Journal of translational medicine 21.

[28] Ishigaki K, Sakaue S, Terao C, Luo Y, Sonehara K, Yamaguchi K, Amariuta T, Too CL, Laufer VA, Scott IC, Viatte S, Takahashi M, Ohmura K, Murasawa A, Hashimoto M, Ito H, Hammoudeh M, Emadi SA, Masri BK, Halabi H, Badsha H, Uthman IW, Wu X, Lin L, Li T, Plant D, Barton A, Orozco G, Verstappen SM, Bowes J, MacGregor AJ, Honda S, Koido M, Tomizuka K, Kamatani Y, Tanaka H, Tanaka E, Suzuki A, Maeda Y, Yamamoto K, Miyawaki S, Xie G, Zhang J, Amos CI, Keystone E, Wolbink G, van der Horst-Bruinsma I, Cui J, Liao KP, Carroll RJ, Lee H-S, Bang S-Y, Siminovitch KA, Vries N de, Alfredsson L, Rantapää-Dahlqvist S, Karlson EW, Bae S-C, Kimberly RP, Edberg JC, Mariette X, Huizinga T, Dieudé P, Schneider M, Kerick M, Denny JC, Matsuda K, Matsuo K, Mimori T, Matsuda F, Fujio K, Tanaka Y, Kumanogoh A, Traylor M, Lewis CM, Eyre S, Xu H, Saxena R, Arayssi T, Kochi Y, Ikari K, Harigai M, Gregersen PK, Yamamoto K, Louis Bridges S, Padyukov L, Martin J, Klareskog L, Okada Y, Raychaudhuri S (2022). Multi-ancestry genome-wide association analyses identify novel genetic mechanisms in rheumatoid arthritis. Nature genetics 54.

[29] Sun J, Wang Y, Folkersen L, Borné Y, Amlien I, Buil A, Orho-Melander M, Børglum AD, Hougaard DM, Melander O, Engström G, Werge T, Lage K (2021). Translating polygenic risk scores for clinical use by estimating the confidence bounds of risk prediction. Nature communications 12.

[30] Breuk A de, Acar IE, Kersten E, Schijvenaars MM, Colijn JM, Haer-Wigman L, Bakker B, Jong S de, Meester-Smoor MA, Verzijden T, Missotten TO, Monés J, Biarnés M, Pauleikhoff D, Hense HW, Silva R, Nunes S, Melo JB, Fauser S, Hoyng CB, Ueffing M, Coenen MJ, Klaver CC, Hollander AI den (2021). Development of a genotype assay for age-related macular degeneration: The EYE-RISK Consortium. Ophthalmology 128.

[31] Kelemen M, Vigorito E, Fachal L, Anderson CA, Wallace C (2024). shaPRS: Leveraging shared genetic effects across traits or ancestries improves accuracy of polygenic scores. American journal of human genetics 111.

[32] Volk RJ, Mendoza TR, Hoover DS, Nishi SP, Choi NJ, Bevers TB (2020). Reliability of self-reported smoking history and its implications for lung cancer screening. Preventive medicine reports 17.

[33] Choi SW, Mak TS-H, O’Reilly PF (2020). Tutorial: a guide to performing polygenic risk score analyses. Nature protocols 15.

[34] Wray NR, Yang J, Hayes BJ, Price AL, Goddard ME, Visscher PM (2013). Pitfalls of predicting complex traits from SNPs. Nature reviews. Genetics 14.

[35] Dogan L, Tanriverdi D, Gungor K (2024). Assessment of vision-related quality of life and depression in patients with age-related macular degeneration. Indian journal of ophthalmology 72.

[36] Casten RJ, Rovner BW (2013). Update on depression and age-related macular degeneration. Current opinion in ophthalmology 24.

